# Spectrum of antihypertensive therapy in South Asians at a tertiary care hospital in Pakistan

**DOI:** 10.1186/1756-0500-4-318

**Published:** 2011-09-01

**Authors:** Aysha Almas, Salik ur Rehman Iqbal, Anabia Ehtamam, Aamir Hameed Khan

**Affiliations:** 1 Department of Medicine, Aga Khan University, Stadium Road, Karachi, Pakistan; 2 Dow University of Health Sciences, Baba-e-Urdu road, Karachi, Pakistan

## Abstract

**Background:**

Despite available guidelines on hypertension (HTN), use of antihypertensives is variable. This study was designed to ascertain frequency of patients on monotherapy and > 1 antihypertensive therapy and also to ascertain proportion of patients on diuretic therapy.

**Methods:**

It was a crossectional study conducted on 1191 adults(age > 18 yrs)hypertensive patients selected by computerized International Classification of Diseases -9-coordination and maintenance (ICD-9-CM) presenting to a tertiary care hospital in Pakistan. Data on demographics, comorbids, type of antihypertensive drug, number of antihypertensive drug and mean duration of antihypertensive drug was recorded over 1.5 year period (2008-09). Blood pressure was recorded on admission. Primary outcome was use of combination therapy and secondary outcome was use of diuretic therapy.

**Results:**

A total of 1191 participants were included. Mean age(SD) was 62.55(12.47) years, 45.3%(540) were males. Diabetes was the most common comorbid; 46.3%(551). Approximately 85% of patients had controlled hypertension. On categorization of anti hypertensive use into 3 categories;41.2%(491) were on monotherapy,32.2%(384) were on 2 drug therapy,26.5%(316) were on ≥3 drug therapy. Among those who were on monotherapy for HTN;34%(167) were on calcium channel blockers,30.10%(148) were on beta blockers, 22.80%(112) were on Angiotensin converting enzyme (ACE) inhibitors,12%(59) were on diuretics and 2.20%(11) were on Angiotensin receptor blockers(ARB). Use of combination antihypertensive therapy was significantly high in patients with ischemic heart disease(IHD)(p < 0.001). Use of diuretics was in 31% (369) patients. Use of diuretics was significantly less in patients with comorbids of diabetes (p 0.02), Chronic kidney disease(CKD)(p 0.003), IHD (p 0.001) respectively

**Conclusion:**

Most patients presenting to our tertiary care center were on combination therapy. Calcium channel blocker is the most common anti hypertensive drug used as monotherapy and betablockers are used as the most common antihypertensive in combination. Only a third of patients were on diuretic as an antihypertensive therapy.

## Background

Hypertension is a leading contributor to the global burden of cardiovascular morbidity and mortality [1]. Despite availability of antihypertensive drugs and recommendation to control hypertension by several bodies, control of hypertension below levels of < 140 systolic and < 90 diastolic is not uniform [2]. Hypertension control has improved from 27.3% in 1988-1994 to 50.1% in 2007-2008 in the United States [3]. However hypertension control rates are barely 6% in countries like Pakistan, China and India [4]. Every, one in three adult aged 40 years and above in Pakistan is hypertensive [5]. Hence the magnitude of the problem for a developing economy like Pakistan is immense.

Several reasons have been highlighted as cause of such high rates of uncontrolled hypertension. Apart from unhealthy lifestyles, lack of awareness about hypertension, distorted public health systems, physicians treating hypertension also lag behind in treating hypertension according to standard guidelines [5,6]. Non compliance to antihypertensive therapy is also a reason for uncontrolled hypertension. Forty three percent patients presenting to outpatient setting at a tertiary care center were not fully compliant in taking antihypertensive medications [7].

The *Seventh Report of the Joint National Committee on Prevention, Detection, Evaluation, and Treatment of High Blood Pressure *(JNC7) recommends diuretic to be used as preferred initial antihypertensive agent alone or in combination [8]. On the other hand excellent clinical trial data suggest that other groups of Antihypertensives like the Angiotensin converting enzyme inhibitor(ACEIs) reduce the complications of hypertension [9,10]. All of the current guidelines suggest that ≥ 1 antihypertensive agent is required in most patients with hypertension to reach BP goals that will effectively reduce the cardiovascular risk [9,11]. Hence there is wide variation in the prescription of antihypertensive medications by physicians all over the world [12]. Four classes of these drugs, including Calcium channel blockers(CCBs), Beta blockers(b-blockers), Angiotensin converting enzyme inhibitors(ACE)/Angiotensin receptor blockers(ARB) and diuretics are the most prescribed antihypertensive drugs class in many parts of the world [13-15].

Data on the use of antihypertensive in individual specialty practices has been reported from the Indo-Asian region. There have been studies advocating the use of ACEIs in stroke patients and use of ARB in normotensive diabetic patients. Hence this data is specific to a certain patient population [16,17]. Currently no robust data on monotherapy and combination therapy are known from this region. Also overall use of diuretics, the first line recommended antihypertensive therapy, along with other antihypertensives from this region is not available. Hence we designed this study to elucidate the spectrum of antihypertensive therapy in South Asian population at a tertiary care center and ascertain frequency of patients on monotherapy and > 1 antihypertensive therapy. Secondary objective was to determine proportion of patients on diuretic as an antihypertensive therapy.

## Methods

### Study design and study population

This was a cross sectional study conducted at the Aga Khan University, Karachi, Pakistan. The Aga Khan University Hospital (AKUH) has 563 beds in operation and provides services to over 50,000 hospitalized patients and to over 600,000 outpatients annually with the help of professional staff and facilities that are among the best in the region. Care is available to all patients in need. Those who are unable to pay for treatment, receive assistance through a variety of subsidies. AKUH is the first hospital in Pakistan and among the first few teaching hospitals in the world to be awarded the prestigious Joint Commission International Accreditation (JCIA) for practicing the highest internationally recognized quality standards in health care. Similarly, the Hospital also holds ISO 9001: 2008 certification for practicing consistent international standards of quality services http://www.aku.edu. Ethical approval from the ethics review committee of the Aga Khan University (letter dated 16-08-2009) was taken for conduct of the study

All adult inpatients(> 18 yrs), over a 1.5 year period(2008-2009), who were known hypertensive (diagnosed on having an average blood pressure of ≥140/90 mm Hg on at least 2 clinic visit)and were on antihypertensive therapy for at least 6 weeks were included [8]. Antihypertensives were prescribed by general physicians, internist and medicine subspecialists. A sample of 1191 consecutive hypertensive patients admitted to the hospital was selected. This sample was drawn using computerized medical record system International Classification of Diseases -9-coordination and maintenance (ICD-9-CM) at health information management system in the hospital. Patients admitted either with primary or secondary diagnosis of hypertension was used for selection through the ICD -9-CM. Those patients who did not have a documented record of the antihypertensive therapy were excluded from the study. All patients gave a general consent on admission; however informed consent was not taken as data was later extracted through ICD-9-CM.

### Study variables and measurements

Primary outcome variable was use of combination antihypertensive therapy. It had three categories; monotherapy, two drug therapy and ≥three drug therapy. Secondary outcome was use of diuretics as an antihypertensive therapy. Data on demographics, co morbid conditions, type of antihypertensive drug, number of antihypertensive drugs and mean duration of antihypertensive drug therapy was recorded by trained data collectors. Records of antihypertensive drugs were extracted from the medical record of the patient. A two way approach was used for this purpose. Anti hypertensives documented on the initial assessment sheets filled at the time of admission was recorded. This was also reconfirmed from the record of the last clinic visit for every patient. In case the clinic visit was at some other hospitals, that prescription was used for the second confirmation. It was assumed that the patients were compliant to the medications written, however no additional measures were used to check compliance. Patients who used an antihypertensive medication with only 1 active ingredient were defined as receiving monotherapy. Those taking more than 1 active ingredient (either in 1 combination pill or in 2 different single pills) were defined as receiving polytherapy [12]. A history of physician-diagnosed diabetes, stroke, ischemic heart disease(IHD), Chronic kidney disease(CKD) as documented in the medical records was noted. Diabetes was defined as fasting plasma glucose ≥126 mg/dl at a prior visit [18]. Stroke was defined clinically as an acute neurologic dysfunction of vascular origin with sudden(within seconds) or at least rapid (within hours)occurrence of symptoms and signs corresponding to the involvement of focal areas in the brain [19]. Ischemic heart disease was diagnosed using WHO definition [20]. CKD was defined as rise in serum creatinine of > 1.2 mg/dl for 3 months [21].

First two readings of blood pressure on admission were recorded. At the time of admission BP in the right arm were recorded using a mercury sphygmomanometer with the individual in the sitting position. Controlled blood pressure was defined as systolic blood pressure (SBP) < 140 mm Hg or diastolic blood pressure(DBP) < 90 mm Hg and uncontrolled blood pressure was defined as SBP > 140 mm Hg and DBP > 90 mm Hg [8]. Any change in antihypertensive medications during inpatient stay was not recorded.

### Statistical analysis

Statistical package for social sciences (SPSS) 17.1 was used for the analysis. Mean and standard deviation was used for quantitative variables and frequency and percentage for qualitative variables. Comparison of qualitative variables was done by chi square test and of quantitative variables by independent sample t test and analysis of variance. p value of < 0.05 was taken as significant.

## Results

A total of 1191 participants were included. The mean age (SD) was 62.5(12.4) years, 45.3%(540) were males. Comorbids conditions and ovearall blood pressure readings are shown in Table 1. Eighty five percent of patients had controlled hypertension.

**Table 1:** Comparison of hypertensive patients on monotherapy versus combination antihypertensive therapy

Characteristics	Overall	Mono therapy	dual therapy	> 3 drugtherapy	P value*
	%(n) N = 1132**	%(n) 41.2(491)	%(n) 32.2(384)	%(n) 14.8(176)	
Age(years) mean (SD)	62.5(12.47)	62.8(12.3)	62.2(12.4)	62.5(12.6)	0.75
Age groups(years) 18-39	3.3(39)	43.6(17)	28.2(11)	28.2(11)	
40-59	33.8(402)	39.1(157)	36.3(146)	24.6(99)	
≥60	62.8(748)	42.2(316)	30.3(227)	27.4(205)	0.33
Gender males	45.3(540)	42.1(216)	32.4(166)	29.3(158)	0.14
**Comorbids conditions**					
Diabetes	46.3(551)	41.2(219)	34.0(181)	27.4(151)	0.62
Chronic kidney disease	8.9(106)	34.6(36)	38.5(40)	28.3(30)	0.25
Ischemic heart disease	34.9(416)	33.6(137)	36.3(148)	31.5(131)	< 0.001
Stroke	11.6(138)	41.4(55)	36.1(48)	25.4(35)	0.9
Chronic liver disease	4.7(56)	41.8(23)	34.5(19)	25(14)	0.91
Systolic Blood pressure mean (SD)	136.2(25)	136.2(23.98)	135.6(25.05)	136.8(27.2)	0.8
Diastolic blood Pressure mean (SD)	77.66(14)	77.7(12.90)	77.4(14.28)	77.7(16.5)	0.9

*p value < 0.05 is significant; it is for the groups(monotherapy,2 drug therapy and ≥3 drug therapy)

**59 patients had missing records for comorbids

### Monotherapy and combination antihypertensive therapy

**Figure 1:**
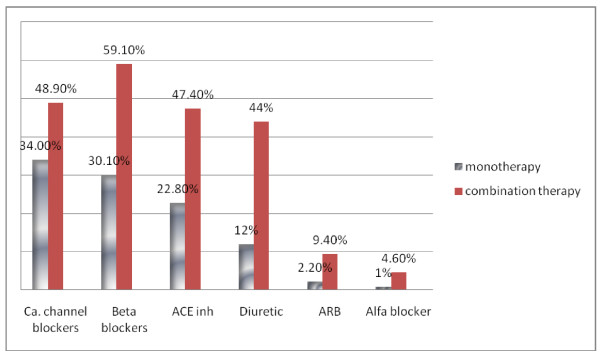
**Distribution of classes of antihypertensive drugs in hypertensive patients on monotherapy and combination therapy**.

On categorization of anti hypertensive into 3 categories; 41.2%(491) were on monotherapy,32.2%(384) were on 2 drug therapy and 26.5%(316) were on ≥3 drug therapy. Comparison is shown in Table 1. Among the three groups, use of mono and dual therapy was significantly increased in patients IHD(p < 0.001). Most diabetics (42%) were on monotherapy. Mean (SD) SBP readings in diabetics were as follows;139.2 (23.7)mm hg for monotherapy,134.9(25.2) mm Hg for 2 drug combination, 136.8(25.1)mm Hg for ≥3 drug combination respectively. Mean (SD) DBP readings in diabetics were as follows;77.7 (11.9)mm hg for monotherapy,76.2(12.8) mm Hg for 2 drug combination, 77.2(15.5)mm Hg for > 3 drug combination respectively. Distribution of classes of antihypertensive drugs in hypertensive patients on monotherapy and combination therapy is shown in Figure 1. Calcium channel blockers were used most commonly as monotherapy; 34%(167) and beta blockers were used as the most common antihypertensive in combination therapy;59.1(41.4)

### Diuretics as an antihypertensive therapy

Thirty one percent patients were on diuretic as antihypertensive therapy. Use of diuretics is significantly decreased in patients with the following comorbids(on diuretic v not on diuretic); diabetes (33% v 66%;p 0.02), CKD (43% v 66%;p 0.003), IHD (37% v 63%;p 0.001) respectively. There was no significant difference in SBP (p 0.10) and DBP (p 0.50) of patients on diuretics compared to not on diuretics.

## Discussion

This data of 1191 Southasian patients at atertiary care center show that, most hypertensive patients were on combination therapy. Patients with IHD, CKD were more likely to be on 2 drug combination antihypertensive therapy. Calcium channel blockers were used as the most common antihypertensive monotherapy and betablockers as most common antihypertensive in combination. Diuretics as monotherapy antihypertensive was used in one third of the patients.

Recent clinical trials suggest that the approach of using monotherapy for the control of hypertension is not likely to be successful in most patients and especially in those with some comorbids conditions. (eg. DM, heart failure). The achievement of BP goal typically requires 2 or more medications in various settings [22-24]. This study shows that two thirds of the patients were on combination therapy. However when combination therapy is replaced by other generic substitutes of the drug it results in poor drug adherence in Pakistani immigrants to Norway [25].

The approach of combination therapy may be theoretically favored by the fact that multiple factors contribute to the hypertension and achieving control of BP with single agent and adding a second agent may lead to better control, acting by complimentary mechanism [11]. Adherence to goal guidelines is better than adherence to treatment guidelines as refelected by the SBP and DBP reported in this study and addition of a third antihypertensive does not decrease the BP further on. Walker reports from the antihypertensive drug prescription trends, a study over a11 year period that drug prescription for antihypertensive combination therapy has increased in all provinces of Canada; Among drug classifications, angiotensin receptor blockers had the largest increase for single-drug therapy and angiotensin-converting enzyme inhibitors-diuretics for combination-drug therapy [26]. However forty percent of diabetics were on monotherapy. Recommended goal of blood pressure (BP) is < 130/80 mm Hg in hypertensives with diabetes mellitus (DM) [27]. This study shows that the target blood pressure as recommended for diabetes was not observed in these patients and this may be attributed to inadequate use of combination therapy. Therefore inadequate use of combination therapy may be one of the reasons of uncontrolled hypertension in diabetics and requires further research. This also highlights the fact that goal attainment is more important than choice of agent.

Calcium channel blockers have emerged as the most common monotherapy used for hypertension, whereas Beta blockers have been used as the most common combination therapy for hypertension. Calcium channel blockers have been the preferred monotherapy in many studies followed by beta blockers and ACE inhibitors [28-31]. ACE inhibitors are found as the most commonly used therapy in other parts [32]. In a cross-sectional screening study conducted in 1000 primary care units considered to be representative of primary care in Turkey most frequently used **antihypertensive **drug class was angiotensin-converting enzyme inhibitors (30.1%), followed by beta-blockers (20.6%), calcium-channel blockers (17.9%), diuretics (15.4%) and angiotensin-receptor blockers (14%) [33]. Hence calcium channel blockers are used as the most common antihypertensive therapy in the Indo Asian region. While calcium channel blockers and beta blockers have surpassed all other antihypertensives as the first line agent used as monotherapy, guidelines all over the world recommend use of low dose thiazide diuretic as the first line drug for essential hypertension [8,34]. Hence, this study indicates that the clinical practice patterns are similar to other parts of the world. This study reports that only 31% patients are on diuretic therapy, which is clearly a low usage compared to what is recommended. Such trends of lower usage of diuretics have also been found in studies conducted in Norway and France [14] Although diuretics are recommended as first line therapy, are ranked as third in total antihypertensive drug utilization [35]. The reason for the low usage in this population could be multiple and needs further research. Firstly physicans tend not to prescribe diuretics due to fear of an adverse electrolyte imbalance, like hyponatremia, which specially in the geriatric population could have detrimental effects [36]. Secondly, promotional activity of pharmaceutical could be such that more marketing is done for the newer antihypertensives like ARB [37].

The strength of this study is the large sample size, and is the first study from this region to report figures on monotherapy, combination therapy and type of antihypertensives in hypertensives. However there are several limitations in our study. Firstly it has limited external validity as the sample is not representative for an entire population. It represents population visiting a standard hospital, hence is not representative of entire Pakistan. Secondly certain amount of recall bias may be involved as information about the antihypertensives was taken from the medical records. Thirdly the crossectional design of the study cannot strongly give a cause and effect association. Fourthly only one blood pressure reading has been taken into account, hence some of the patients may have been misclassified as controlled hypertensives. Also compliance and cost of antihypertensives was not assessed for each patient.

## Conclusion

Most patients presenting to our tertiary care center were on combination therapy. Calcium channel blocker is the most commonly used anti hypertensive drug used as monotherapy while betablockers is used as the most common combination therapy. Diuretics use in hypertensive patients is low and not keeping with the recommended guidelines.

## Competing interests

The authors declare that they have no competing interests.

## Authors' contributions

AA generated the idea, designed the study and was involved in analysis and first draft. SRI and AE were involved in the data management and analysis. AHK was involved in designing, proof reading and reviewing the draft. All authors read and approved the final manuscript
